# Swift Heavy Ion-Induced Chemistry of CH_3_CN Ices at 10 and 80 K

**DOI:** 10.1021/acsearthspacechem.5c00379

**Published:** 2026-02-13

**Authors:** Ana Lucia Ferreira de Barros, Cintia Aparecida Pires da Costa, Yahia Murhej, Raghunandanan Sreeja, Davi Viana Doreste, Enio Frota da Silveira, Philippe Bouduch, Hermann Rothard, Matteo Michielan, Daniela Ascenzi, Alicja Domaracka

**Affiliations:** † Departamento de Física, 74351 Centro Federal de Educação Tecnológica Celso Suckow da Fonseca, Av. Maracanã 229, Rio de Janeiro, Rio de Janeiro 20271-110, Brazil; ‡ Dipartimento di Fisica, 19034 Università di Trento, Trento 38123, Italy; § 131977 Normandie Univ., ENSICAEN, UNICAEN, CEA, CNRS, CIMAP, Caen 14000, France; ∥ Departamento de Física, 28099 Pontifícia Universidade Católica Do Rio de Janeiro, Rua Marquês de São Vicente 225, Rio de Janeiro, Rio de Janeiro 22451-900 Brazil

**Keywords:** astrochemistry, methods, solid state, astronomical instru mentation, methods, techniques, spectroscopic interstellar medium (ISM), nebulae, cosmic rays

## Abstract

Acetonitrile (CH_3_CN) is a key nitrogen-bearing molecule detected in a variety of astrophysical environments and is considered a potential precursor of prebiotic compounds. The study aimed to investigate the stability and radiation chemistry of ^56^Fe^10+^ ions at the Grand Accélérateur National d’Ions Lourds (GANIL). In situ FTIR spectroscopy revealed efficient molecular destruction accompanied by the formation of several nitrogen-bearing species, including HCN, H_2_C=C=NH, CH_2_CHNC, CH_3_CH=NH, H_2_C=NH, NCCN, and NH_3_ with possible contributions from the C–H such as CH_4_ and C–H stretching of HC_3_N and, to a lesser extent, the N–H stretch of ketenimine (H_2_C=C=NH). The apparent destruction cross section of CH_3_CN was found to be (2.3 ± 0.8) × 10^–12^ cm^2^ at 10 K and (5.6 ± 1.0)×10^–12^ cm^2^ at 80 K, indicating more extensive radiolytic processing at higher temperatures. Enhanced radical mobility at 80 K promotes hydrogenation and polymerization, leading to a refractory C–N–rich residue, whereas at 10 K intramolecular isomerization dominates. These results demonstrate that swift heavy-ion irradiation of nitrile ices efficiently produces small nitriles, isonitriles, and polyimines of astrochemical interest, supporting the role of CH_3_CN as a hidden precursor to prebiotic organic matter in interstellar and planetary ices.

## Graphical abstract

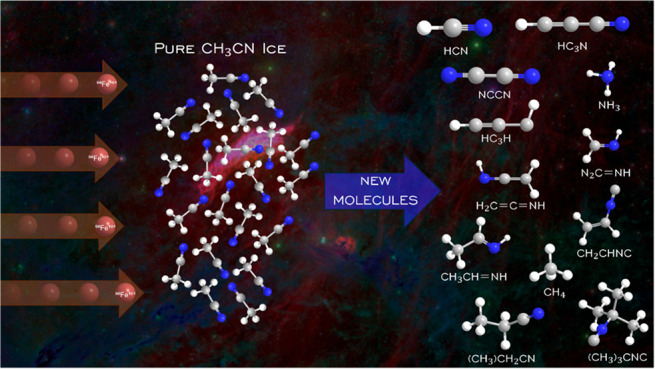

## Introduction

Nitriles are key molecular species in astrochemistry, having been detected in the interstellar medium (ISM), in comets, and in planetary atmospheres such as Titan’s dense atmosphere.^1–3^ Among them, acetonitrile (CH_3_CN, methyl cyanide) is the simplest organic nitrile and a prototypical nitrogen-bearing complex organic molecule (COM). Its presence has been confirmed in the gas phase of Titan^1^ in cometary comae,^4^ and in the ISM where nitriles compose one of the largest molecular families identified so far.^2,5^ Due to its relative high abundance and reactivity, CH_3_CN is considered a fundamental precursor in the pathways leading to amino acids and other prebiotic species.^4,6^

Laboratory studies have shown that nitrile-containing ices subjected to energetic processing undergo extensive chemical transformations. Ion irradiation, UV photolysis, and X-ray exposure induce isomerization, fragmentation, and polymerization processes, yielding products such as isonitriles, ketenimines, cyanate ions, and amino acid precursors.^4,7,8^ For acetonitrile, irradiation at cryogenic temperatures leads to the formation of radicals (H_2_CCN, H_2_CNC),^9^ volatile nitriles including acrylonitrile and propionitrile,^7,10^ and, after hydrolysis, amino acids similar to those found in carbonaceous chondrites.^4^ These results reinforce the hypothesis that CH_3_CN-containing ices contribute to the molecular complexity observed in astrophysical environments, particularly in regions exposed to cosmic rays and other ionizing radiation.

The infrared spectral properties of CH_3_CN ices have been extensively characterized under laboratory conditions. Early studies reported optical constants and integrated absorption coefficients of crystalline nitriles at low temperatures, relevant for the outer Solar System.^1^ More recent works clarified the crystalline phases of CH_3_CN and its congeners,^11^ reported IR band strengths for amorphous and crystalline forms,^5^ and highlighted how ice structure and temperature control reactivity. These data are indispensable for interpreting remote IR observations (Voyager, Cassini, JWST) and for quantifying molecular abundances in extraterrestrial environments.

Despite these advances, the role of heavy ions remains underexplored. Most irradiation studies employed light projectiles such as protons or electrons,^6^ whereas swift heavy ions very abundant in dense interstellar regions and planetary magnetospheres—deposit large electronic energy densities along their tracks, efficiently inducing molecular dissociation and complex chemistry. Experiments on other nitrile ices under heavy-ion irradiation have demonstrated significant production of CN-bearing species and prebiotic molecules,^4,7^ but systematic work on pure CH_3_CN at astrophysical temperatures is scarce.

Here, we report a laboratory investigation of the radiolysis of pure acetonitrile ices by swift ^56^Fe^10+^ ions at 10 and 80 K, performed at the Grand Accélérateur National d’Ions Lourds (GANIL, Caen, France). These two temperatures simulate, respectively, the deeply frozen conditions of dense interstellar clouds and the warmer environments of outer Solar System bodies such as Titan’s surface.^1,11^ By combining in situ Fourier-transform infrared (FTIR) spectroscopy with controlled ion fluences, we determine the destruction cross sections of CH_3_CN, identify stable and transient products, and evaluate the influence of temperature on radiation-driven chemistry. The results provide new insights into the potential of acetonitrile ices to act as reservoirs of prebiotic precursors in astrophysical environments exposed to heavy-ion irradiation.

## Experimental Procedures

The experiments were performed at the IGLIAS facility (French acronym for “Irradiation of astrophysical ices”) located at CIMAP-CIRIL, using the IRRSUD beamline of the Grand Accélérateur National d’Ions Lourds (GANIL, Caen, France).^12^ This beamline provides stable and well-characterized heavy-ion beams with controlled fluence and energy, making it suitable for simulating the high-LET (linear energy transfer) conditions experienced by astrophysical ices under cosmic-ray bombardment.^13–15^ In this work, swift ^56^Fe^10+^ ions were employed as projectiles to irradiate thin films of acetonitrile ice.

### Sample Preparation

Thin films of pure CH_3_CN were grown by vapor deposition onto a ZnSe optical substrate mounted on a cold finger in an ultrahigh vacuum chamber with a base pressure below 5 × 10^–10^ mbar. The substrate, a 2 mm thick, 20 mm diameter optical-grade ZnSe window, was chosen for its excellent transmission in the mid-infrared region. High-purity liquid acetonitrile (Sigma-Aldrich, 99.9%) was purified by several freeze–pump–thaw cycles at liquid nitrogen temperature to remove dissolved gases and volatile contaminants. The vapor was introduced through a deposition line positioned 15 mm in front of the substrate, ensuring homogeneous film growth.

The substrate temperature was controlled using a calibrated silicon diode sensor in thermal contact with the sample holder, with stability better than ±0.5 K. Films were deposited at 10 K and, in separate experiments, at 80 K to simulate, respectively, dense interstellar/circumstellar environments and surface conditions of outer Solar System bodies. The reproducibility of band positions, profiles, and integrated absorbances across independent preparations confirmed film homogeneity.

### Infrared Spectroscopy

Fourier-transform infrared (FTIR) spectra were recorded in transmission mode covering the 5000–600 cm^–1^ range, with 1 cm^–1^ resolution, and averaging over 70 scans. Prior to each deposition the background spectrum, acquired including a clean substrate at the same vacuum and temperature conditions, was used for baseline subtraction.^16^ The evolution of infrared band areas with ion fluence is necessary to monitor molecular destruction and product formation.

The initial column density *N*_0_ (molecules cm^–2^) of CH_3_CN was determined from the absorbance of the CN stretching mode at 2252 cm^–1^ using the Beer–Lambert relation:

1 $$N_{0}=\frac{1}{A}\int \tau \left(\nu \right)\;d\nu$$

where τ­(ν) is the optical depth as a function of wavenumber and *A*-value (*A*_*v*_) is the band strength. For CH_3_CN, the adopted band strength for the mentioned mode is *A*_*v*_ = 2.2 × 10^–18^ cm molecule^–1^.^6,7,17^

### Irradiation Conditions

Ion irradiations were performed at normal incidence using swift Fe beams delivered by the IRRSUD beamline. The ion kinetic energy was set to 0.71 MeV per nucleon, corresponding to a total energy of approximately 40 MeV for ^56^Fe^10+^ ions. According to SRIM simulations, the projected ion range in solid CH_3_CN under these conditions lies between 4 and 40 μm, depending on the assumed electronic stopping power regime of 10^3^–10^4^ keV μm^–1^, with a representative intermediate value of ∼8 μm. This penetration depth exceeds the thickness of the deposited ice films (approximately 3–4 μm) by a factor of at least two, ensuring full ion traversal and nearly homogeneous energy deposition throughout the irradiated volume. The high electronic stopping power combined with complete film penetration provides a highly uniform irradiation environment, efficiently triggering radiolytic chemistry and enabling the formation of complex nitrogen-bearing organic species within the Fe ion tracks.

### Ice Thickness Determination

The thickness η of the CH_3_CN films was estimated from the initial column density *N*_0_ using

2 $$\eta =\frac{N_{0}\;M{\;10}^{4}}{\rho {\;N}_{A}}$$

where η is in μm, *M* is the molar mass of CH_3_CN (41.05 g mol^–1^), ρ is the density of amorphous CH_3_CN ice (0.77 g cm^–3^ at cryogenic temperatures^11^), and *N*_*A*_ is Avogadro’s constant. For the present experiments, by eq 2, the column densities ranged from 4 × 10^17^ molecules cm^–2^ for 10 K to 10 × 10^17^ molecules cm^–2^ for 80 K, corresponding to film thicknesses of approximately 3–4 μm. These values are compatible with the calculated ion ranges, confirming that (i) the energetic Fe projectiles fully traversed the prepared ices and (ii) the energy was deposited approximately uniform throughout the films.

## Results and Discussion

### Infrared Spectral Evolution under Fe-Ion Irradiation at 10 and 80 K

Heavy-ion bombardment of the CH_3_CN ice causes dramatic changes in the IR spectrum, indicating efficient molecular destruction and new species formation. Figures 1 and 2 illustrate the evolution of the mid-IR absorption bands of a pure acetonitrile ice during ^56^Fe^10+^ irradiation. The initially deposited CH_3_CN at 10 K exhibits its characteristic vibrational features:^2,5^ a strong ν_*CN*_ band of the nitrile group at ∼2252 cm^–1^, the $\nu_{CH_{3}}$ symmetric stretch at ∼2941 cm^–1^ and ∼3002 cm^–1^, the $\nu_{CH_{3}}$ deformation modes near 1375, 1410, and 1450 cm^–1^, and the CH_3_ rocking mode at ∼1040 cm^–1^. At 80 K, these CH_3_CN bands appear slightly red-shifted and narrowed due to temperature-dependent matrix effects, in agreement with prior studies.^11^ High-resolution infrared data obtained from X-ray irradiation of acetonitrile isolated in noble-gas matrices further support these assignments, providing benchmark band positions for isolated CH_3_CN and its primary dissociation products and helping to disentangle intrinsic molecular features from temperature- and environment-induced effects.^18,19^

**Figure 1:**
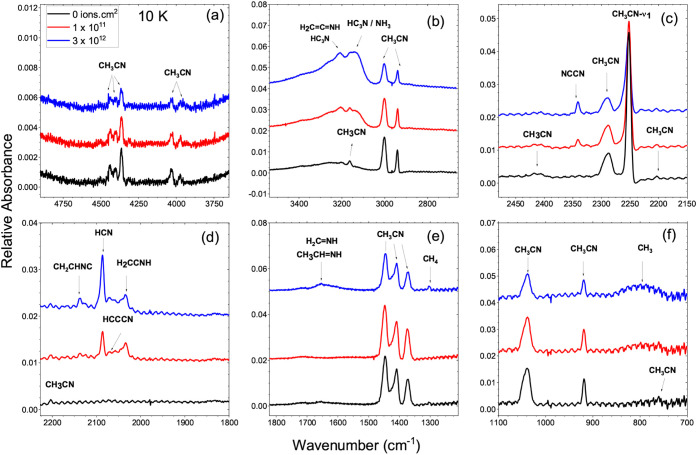
Infrared spectra of CH_3_CN ice at 10 K before and after irradiation at fluences of 0 (black), 1 × 10^11^ (red), and 3 × 10^12^ ions cm^–2^ (blue). Panels (a–f) show different spectral regions highlighting the evolution of CH_3_CN bands and the formation of radiolysis products such as HCN, H_2_C=NH, and HC_3_N. Spectra are vertically shifted for clarity.

**Figure 2:**
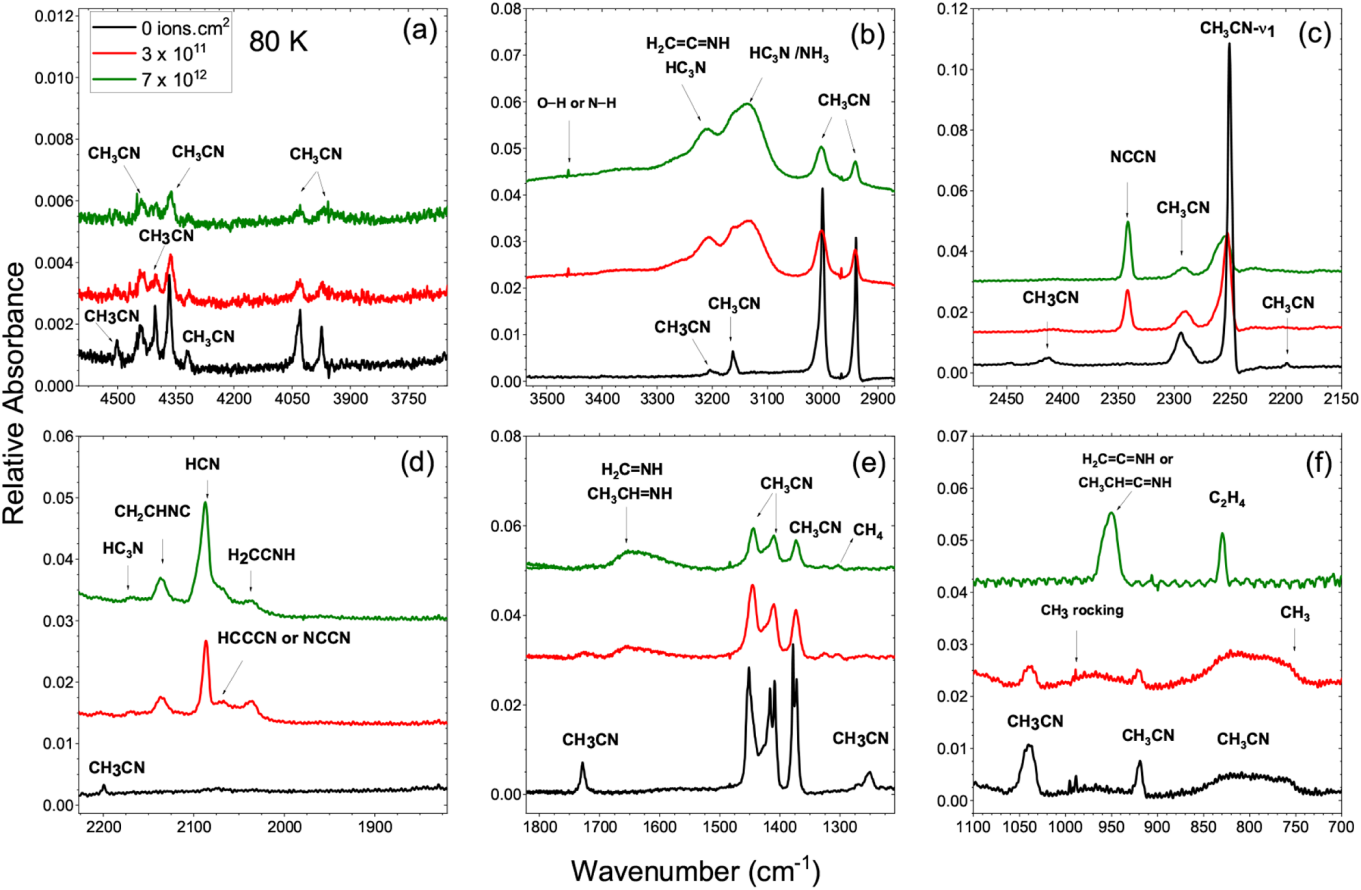
Infrared spectra of CH_3_CN ice at 80 K before and after irradiation at fluences of 0 (black), 3 × 10^11^ (red), and 7 × 10^12^ ions cm^–2^ (green). Panels (a–f) display different spectral regions, showing the evolution of CH_3_CN features and the appearance of radiolysis products such as HCN, H_2_C = NH, and HC_3_N. Spectra are vertically shifted for clarity.

The initial column density *N*_0_ of CH_3_CN was determined from the integrated absorbance of the CN stretch band using its band strength *A*(CN) ≈ 2.2 × 10^–18^ cm molecule^–1^.^6,7^ This yields *N*_0_ ∼ 3.5 × 10^17^ molecules cm^–2^ for the as-deposited ice, corresponding to an ice film thickness on the order of ∼1 μm (assuming a solid density ρ = 0.77 g cm^–3^ for amorphous CH_3_CN).^11^

As can be seen in Figure 1 at 10 K, CH_3_CN ice is mainly amorphous, with broad and less-resolved infrared bands. Each panel highlights the infrared spectra at distinct spectral regions: (a) 4800–3700 cm^–1^, showing weak overtone and combination features; (b) 3600–2700 cm^–1^, corresponding to N–H and C–H stretching modes; (c) 2450–2150 cm^–1^, displaying the C≡N stretching of CH_3_CN and newly formed nitriles; (d) 2200–1800 cm^–1^, where products such as HCN, H_2_C=NH, and HC_3_N are observed; (e) 1800–1200 cm^–1^, encompassing C–H bending and C=N stretching regions; and (f) 1100–700 cm^–1^, showing CH_3_ rocking and wagging modes. The progressive appearance of new bands indicates the radiolytic formation of secondary species such as HCN, H_2_C=NH, and HC_3_N. The strongest feature, the C≡N stretching mode, appears near 2252 cm^–1^, with a relatively wide profile. CH_3_ deformation and rocking bands (1374–1040 cm^–1^) are also broad and overlapping.

At 80 K (Figure 2), the ice undergoes partial crystallization into a more ordered phase. Consequently, the vibrational bands become narrower and more intense, often showing small redshifts (1–5 cm^–1^). The C≡N stretching band shifts to ∼2249–2254 cm^–1^ and becomes sharper, while CH_3_ bending and rocking modes are better resolved. These spectral changes are consistent with the amorphous–crystalline transition of CH_3_CN ices previously reported in laboratory studies.^5–7,11^ Similar narrowing and splitting of vibrational features have been observed for acetonitrile isolated in noble-gas matrices at cryogenic temperatures, where matrix isolation suppresses intermolecular coupling and highlights site-specific environments.^18^ In contrast, the present neat-ice experiments probe collective solid-state effects, including partial amorphization induced by high-LET ion tracks.

Figure 2 presents the infrared spectra of CH_3_CN ice at 80 K before and after irradiation as a function of ion fluence. At this temperature, the CH_3_CN ice is partially crystalline, exhibiting sharper and better-resolved absorption bands compared with the amorphous sample spectra at 10 K. Each panel highlights a distinct spectral region: (a) 4800–3700 cm^–1^, showing weak overtone and combination features; (b) 3600–2700 cm^–1^, corresponding to N–H and C–H stretching modes; (c) 2450–2100 cm^–1^, dominated by the C≡N stretching mode of CH_3_CN and newly formed nitriles; (d) 2200–1800 cm^–1^, where HCN, H_2_C=NH, and HC_3_N are observed; (e) 1800–1200 cm^–1^, encompassing C–H bending and C=N stretching regions; and (f) 1100–700 cm^–1^, displaying CH_3_ rocking and wagging modes.

Table 1 compares the CH_3_CN ice spectrum acquired at 10 K with spectra obtained at 80 K. More pronounced vibrational band splitting is observed at 80 K, especially in the CH_3_ deformation region (1370–1450 cm^–1^) and in the C≡N stretching region near 2250 cm^–1^, where a new shoulder emerges at 2294 cm^–1^. These spectral changes reflect the onset of crystallization and the presence of inequivalent molecular environments within the ordered CH_3_CN lattice. Additionally, part of the initially crystalline or partially ordered ice becomes amorphous during swift Fe-ion irradiation (as illustrated in Figure 2c and Figure 2e), causing absorption bands to widen and undergo small red shifts. At 80 K, CH_3_CN ice is partially crystalline prior to irradiation. Heavy-ion impact induces local amorphization through electronic excitation along ion tracks, leading to a mixed ice morphology during irradiation. The coexistence of ordered and disordered domains affects both the observed spectral profiles and the resulting chemistry.

**Table 1:** Observed Band Positions of CH_3_CN Ice at 10 and 80 K, Vibrational Assignments, and Adopted Band Strengths *A*-Values for 10 K^5^

10 K (cm^–1^)	80 K (cm^–1^)	Mode/Assignment	*A*_*v*_ x 10^–18^ cm molecule^–1^
760.2 ^6–8^	–	ν_8_(CCN bend, out-of-plane)	–
919.7 ^5–8^	918.2	CH_3_ wag/rock (libration/rocking of methyl)	0.35 ^5^
1039.1 ^5–8^	1039.6	CH_3_ rock (umbrella/rocking)	1.6 ^5^
–	1255.5	ν_7_ (CH_3_ rock) + C–C skeletal deformation	–
1374.3 ^5–8^	1372.1/1378.3	CH_3_ symmetric deformation (δ_*s*_)	1.2 ^5^
1409.2 ^5–8^	1408.7/1416.4	CH_3_ antisymmetric deformation (δ_*as*_)	1.9 ^5^
1446.6 ^5–8^	1450.1	CH_3_ scissoring/combination (δ CH_3_)	2.9 ^5^
–	1728.5 ^7,8,20^	overtone/combination (ν(C=N))	–
–	2199.4 ^2,6,11^	ν(C≡N) stretch (secondary nitriles)	–
2252.1 ^2,5–8^	2250.7	C≡N stretching (ν CN, principal)	1.9 ^5^ 2.2 ^6,7^
2288.2 ^5–8^	2294.4	shoulder/perturbed ν(C≡N)	0.62 ^5^
2412.0 ^6–8^	2412.9	overtone/combination (anharmonic)	–
2631.6 ^6–8^	2633.4	overtone/combination (anharmonic)	–
2941.4 ^5–8^	2939.9	ν(C–H) symmetric stretch	0.53 ^5^
3001.9 ^5–8^	3000.9	C–H stretching region (ν CH_3_)	1.5 ^5^
3163.2 ^6–8^	3162.5	ν_2_ + ν_4_ combination in CH stretching	–
3971.2 ^6,8^	3973.2	weak overtone (CH region, ν_1_ + ν_7_)	–
4032.9 ^6^	4030.8	ν(C≡N) + CH_3_ bending combination	–
–	4317.7 ^2,5,11^	ν(C≡N) + CH_3_ bending combination	–
4362.8 ^2,6^	4363.8	overtone/combination ν(C≡N) (2ν_2_)	
4403.6 ^6^	4403.1	combination ν_1_(CH_3_ sym.) + CH_3_ rocking	–
4440.7	4441 ^2,5,11^	overtone/2ν(C≡N)	–

Unlike matrix-isolation experiments, heavy-ion irradiation induces amorphization through dense electronic excitation and localized energy deposition, leading to substantial restructuring of the solid. The emergence of this disordered component ensures full energy deposition across the film and enables a direct comparison with radiation-processed amorphous solids. New absorption features associated with radiolysis products such as HCN, H_2_C=NH, and HC_3_N increase with fluence, indicating efficient molecular fragmentation followed by radical recombination and chain-growth chemistry promoted by molecular excitations around the ion tracks. At 80 K, partial structural ordering and increased molecular mobility do not improve spectral resolution but alter band intensities and selection rules, allowing weak overtones and combination bands to become detectable compared to 10 K.

At 80 K, CH_3_CN ice is initially partially crystalline, but heavy-ion irradiation induces local amorphization through dense electronic excitation and transient thermal spikes along ion tracks. As a result, crystalline and radiation-damaged amorphous domains coexist during irradiation. While an overall narrowing of vibrational bands is not expected, this mixed morphology and the finite molecular mobility at 80 K modify band intensities and selection rules, allowing weak overtones and combination bands to become detectable.

The enhanced spectral complexity observed at 80 K reflects the increased mobility of H, CN, and other small radical fragments, which enables secondary reactions such as hydrogenation, radical–radical recombination, and acid–base processes beyond the initial dissociation sites. In contrast, at 10 K radiolysis products remain largely trapped within their original molecular cages. Under these conditions, chemistry is dominated by geminal recombination,^7,21^ limited intramolecular rearrangements, and frequent reformation of the parent CH_3_CN molecule, resulting in a much more restricted chemical diversification.

At 10 K and after irradiation (Figure 3 top), three additional features can be assigned as follows. In Figure 3a, 3212 cm^–1^ band corresponds to the N–H stretching of H_2_C=C=NH slightly red-shifted by the matrix; in Figure 3c, the very weak band at 1343 cm^–1^ refers to the CH_2_ deformation/wagging mode of H_2_C=NH, and the 1303 cm^–1^ one to the ν_4_ deformation of radiolytically formed CH_4_.

**Figure 3:**
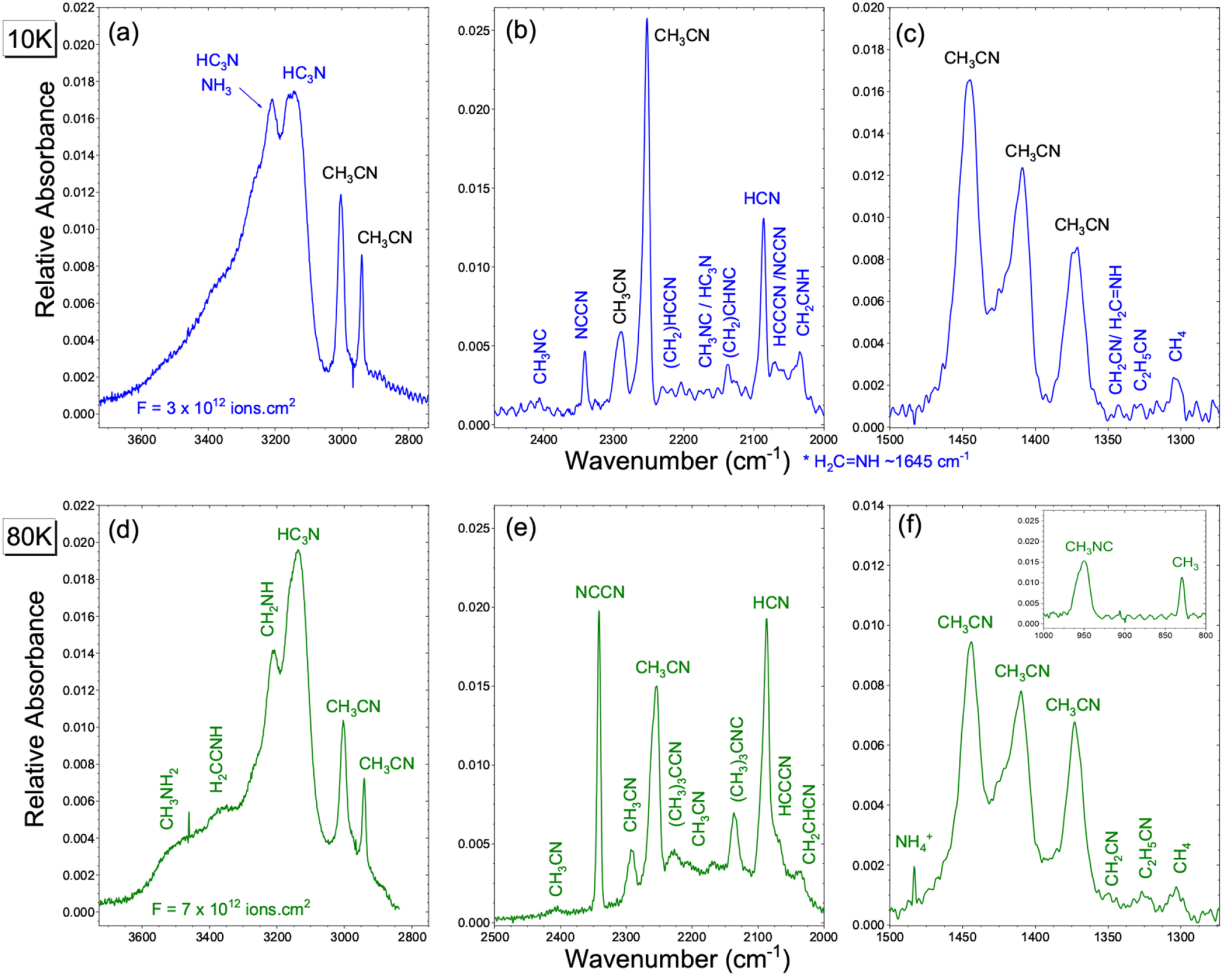
Infrared spectra of CH_3_CN ice at 10 K (a–c, blue) and 80 K (d–f, green) after ^56^Fe^10+^ irradiation at fluences of 3 × 10^12^ and 7 × 10^12^ ions cm^–2^, respectively. Panels show the main vibrational regions of CH_3_CN and its radiolysis products. Sharper and split bands at 80 K indicate partial crystallization, while new features from CH_3_NH_2_ (displayed in (e)) and ${\mathrm{NH}}_{4}^{+}$ (displayed in (f)) reveal enhanced radiation-induced chemistry at elevated temperature. The inset at 80 K shows the 1000–800 cm^–1^ region; the band near ∼ 940 cm^–1^ is CH_3_NC, while the broader feature around ∼830 cm^–1^ arises from CH_3_ rocking modes of CH_3_CN.

At 80 K, Figure 3 (bottom), three additional features not detected at 10 K are observed at ∼3518, ∼3372, and ∼1483 cm^–1^. The weak absorption near ∼3518 cm^–1^ is consistent with either the free N–H stretching of a primary amine (e.g., CH_3_NH_2_) or with weakly hydrogen-bonded NH_3_. Owing to band overlap and low intensity, the presence of CH_3_NH_2_ cannot be conclusively confirmed under our experimental conditions. The feature at ∼3372 cm^–1^ (Figure 3d) corresponds to the N–H stretching of hydrogen-bonded NH_3_ or an amine/imine environment, which becomes more prominent upon partial crystallization.

The ∼1483 cm^–1^ band matches the ν_4_ bending mode of ${\mathrm{NH}}_{4}^{+}$ , indicating acid–base chemistry (NH_3_ + HCN → ${\mathrm{NH}}_{4}^{+}$ + CN^–^) enabled by the higher molecular mobility at 80 K, as seen in Figure 3f. The appearance of this band only at elevated temperature, together with the concurrent growth of NH_3_-related N–H stretching features, strongly supports its assignment to ${\mathrm{NH}}_{4}^{+}$ rather than neutral amines. Similar ammonium signatures have been reported for irradiated nitrogen-bearing ices where proton transfer becomes efficient once diffusion barriers are partially overcome. Table 2 shows the comparison of the main bands of CH_3_CN ice observed at 10 and 80 K.

**Table 2:** Comparison of Main Infrared Band Positions of CH_3_CN Ice at 10 and 80 K^a^

Mode/Assignment	10 K (cm^–1^)	80 K (cm^–1^)	Observation
C–H stretching (CH_3_)	∼3002	∼3001	Slight redshift, narrower
C≡N stretching	2252	2251	Sharper at 80 K (crystalline phase)
CH_3_ deformation (sym.)	1374	1372–1378	Narrower at 80 K
CH_3_ deformation (asym.)	1409	1409–1416	Redshift, sharpening
CH_3_ rocking	1039	1040	More resolved at 80 K
Lattice/comb. modes	broad	sharper	Features after crystallization

a Values and spectral behaviorare consistent with previous works.^5–7,11^

### Infrared Band Evolution of CH_3_CN at 10 and 80 K

Figures 4 and 5 show the evolution of the column densities of selected CH_3_CN infrared bands as a function of ion fluence at 10 and 80 K, respectively. At 10 K (Figure 4), all major CH_3_CN vibrational modes progressively decrease in intensity with increasing fluence, confirming the molecular destruction induced by Fe ion bombardment. The most persistent features correspond to the C≡N stretching mode (2252 cm^–1^), the CH_3_ rocking mode (1039 cm^–1^), and the CH_3_ deformation modes (1374, 1410, 1447 cm^–1^). Their exponential-like decay with fluence reflects the typical first-order kinetics of radiolytic dissociation in condensed ices.

**Figure 4:**
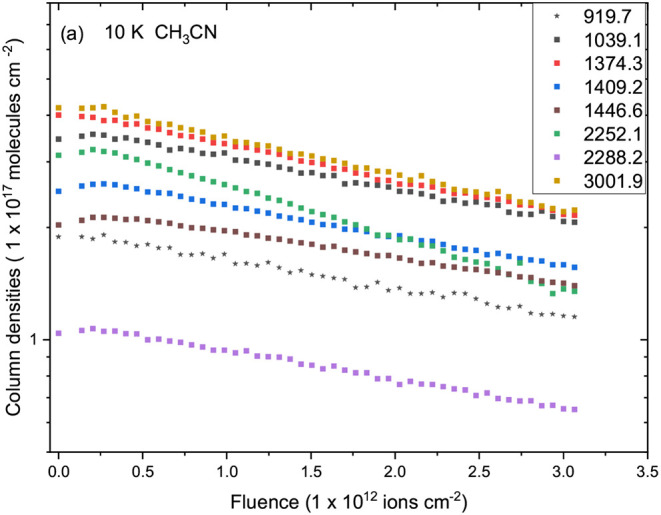
Evolution of the column densities of selected infrared bands of CH_3_CN ice at 10 K as a function of Fe ion fluence. Each symbol represents a vibrational mode listed in the legend. A gradual decrease in the main CH_3_CN features indicates molecular destruction by ion irradiation.

**Figure 5:**
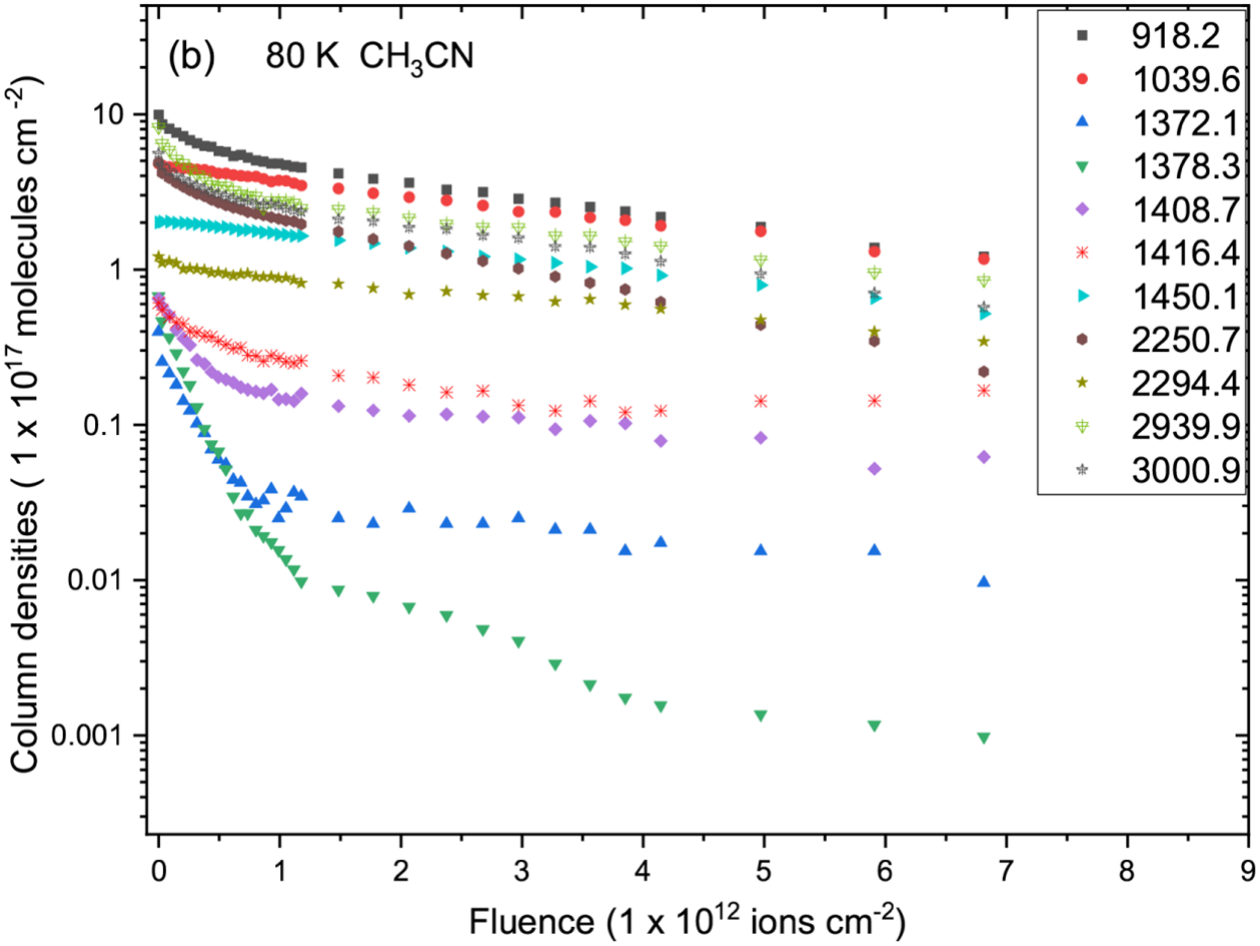
Evolution of the column densities of selected infrared bands of CH_3_CN ice at 80 K as a function of Fe ion fluence. Compared to 10 K, a greater number of vibrational modes and new features appear at 80 K, reflecting enhanced radical diffusion and secondary reactions.

In contrast, at 80 K (Figure 5), the CH_3_CN destruction is accompanied by a diversified spectral evolution. While the parent bands remain initially visible (918, 1450, and 2250 cm^–1^), the higher temperature enhances molecular diffusion and radical recombination, giving rise to numerous new absorptions at 2294, 2412, 2633, 2939, and 3162 cm^–1^. These features correspond to overtone and combination modes, as well as to vibrational signatures of newly formed products—including imines, isonitriles, and unsaturated nitriles. The increased mobility at 80 K facilitates structural rearrangements and secondary chemistry, producing overlapping absorption bands and a more complex spectral pattern compared to the 10 K experiment.

Overall, both temperatures exhibit clear evidence of CH_3_CN destruction under ion irradiation, but the 80 K spectra reveal an active chemical network leading to molecular rearrangements characterizing the emergence of new nitrogen-bearing products. SRIM-code estimates combined with IR tracking show that the crystalline phase of CH_3_CN undergoes progressive lattice damage and becomes predominantly amorphous at fluences above 2 × 10^12^ ions cm^–2^ for swift ^56^Fe^10+^ irradiation at normal incidence, identified experimentally by the full collapse of CN band splitting into a single broad red-shifted C≡N profile. In this high-fluence regime, absorption bands widen and shift slightly to lower wavenumbers, marking the loss of long-range lattice order and enabling direct comparison with literature reports on irradiated amorphous CH_3_CN ices. The temperature-dependent behavior underscores the role of solid-state thermal mobility in driving nitrile destruction, radical diffusion, and ion-track-induced nonthermal rearrangements. Moderate molecular mobility at 80 K enhances radical-driven chemistry within Fe-ion tracks, feeding a condensed-phase reaction network that supports the buildup of complex nitrogen-bearing organic products in radiation-processed nitrile-rich astrophysical ice analogs.

### Identification of Radiolysis Products after Irradiation

#### Hydrogen Cyanide (HCN)

The most prominent new feature appears at ∼2085 cm^–1^, which we assign to the C≡N stretching vibration of hydrogen cyanide (HCN). This band grows steadily with fluence, indicating that HCN is a major radiolysis product. The peak position (2086 cm^–1^ at 80 K) matches literature values for solid HCN (2085–2090 cm^–1^).^4,8^ HCN formation from CH_3_CN suggests rupture of the C–C bond and recombination of the liberated −CN fragment with hydrogen. Indeed, UV photolysis of CH_3_CN is known to produce CN radicals that readily form HCN by abstracting H atoms.^7,22^

In the current experiments, abundant free H atoms are generated by bond cleavages (see below), many of them combine with CN to yield HCN. Using an IR band strength *A*(HCN) ≈ 5 × 10^–18^ cm molecule^–1^,^2^ we estimate the HCN column density after the highest fluence (3 × 10^12^ ions cm^–2^) to be about 4 × 10^16^ cm^–2^ (tens of monolayers). This corresponds to a yield of roughly several HCN molecules produced per incident Fe ion (considering the ∼10^13^ cm^–2^ fluence range), underscoring the high efficiency of heavy-ion chemistry.

HCN is a relevant molecule in astrophysics, observed in many environments (ISM clouds, comets, Titan’s atmosphere). The current results reinforce that CH_3_CN is an efficient solid-state source of HCN under energetic processing.^7^ Notably, we did not detect any separate absorption attributable to its tautomer HNC (hydrogen isocyanide) in the irradiated ice. In the solid state, the CN stretching mode of HNC is expected near ∼2060–2070 cm^–1^, distinctly lower than the HCN ν­(C≡N) band at ∼2095–2100 cm^–1^.^2,7^ Gas-phase HNC is well-known in cometary comae and cold interstellar clouds; however, in laboratory ices its formation is generally suppressed.^7^ In the presence of H_2_O or other oxygen-bearing species, CN-containing ices preferentially convert HCN into OCN^–^, further decreasing the likelihood of observable HNC production.^23,24^

For the pure CH_3_CN ices, any nascent HNC produced by intramolecular rearrangement of HCN likely rapidly isomerizes back to the more stable HCN or is scavenged by remaining radicals. This is consistent with previous ion irradiation studies of pure nitriles, which found little to no HNC in the IR spectra.^7^ We conclude that HCN is the dominant C–N–H species formed, with negligible trapped HNC in the solid phase under our conditions (in accord with Gerakines et al. 2004’s finding that HNC is essentially absent in photolyzed HCN ices).^24^

#### Nitrile Isomers: CH_3_NC and H_2_C=C=NH

In addition to the formation of fragment products (HCN, CH_4_), heavy-ion irradiation may induce intramolecular rearrangements of CH_3_CN, yielding its structural isomers. Two of such isomers are methyl isocyanide (CH_3_NC) and ketenimine (H_2_C=C=NH), which have been observed in prior CH_3_CN photolysis and radiolysis experiments and are believed to form via single-molecule reconfigurations (tautomerization) of the acetonitrile backbone.^7^

Isoacetonitrile (CH_3_NC) is expected to be a major radiolysis product of CH_3_CN and was unambiguously identified in soft X-ray irradiation experiments performed under matrix-isolation conditions by Kameneva et al.^18^ and Volosatova et al.^19^ In these studies, CH_3_NC formation was confirmed by its strong absorption near 2160 cm^–1^ and an additional characteristic band around 940 cm^–1^.^18,19^ In the present experiments, we examined both spectral regions. A feature near 2160 cm^–1^ is observed at intermediate fluences and is consistent with the C≡N stretching mode of CH_3_NC; however, its assignment in neat CH_3_CN ice is difficult because of the overlap with other CN-bearing species, baseline uncertainty, and band broadening intrinsic to compact condensed films.

The low wavenumber diagnostic region of isoacetonitrile was also examined and is shown in the inset of Figure 3f at 80 K. The band near ∼940 cm^–1^, which is characteristic of CH_3_NC, becomes more clearly identifiable when this spectral window is isolated; however, in neat ice experiments this region is still affected at high fluences by reduced signal-to-noise ratio and partial overlap with other irradiation products and lattice-related modes. Although the observed features are compatible with isoacetonitrile formation, an unambiguous assignment cannot always be made based on this region alone. We therefore adopt a conservative assignment strategy and refer to CH_3_NC as “consistent with” or “tentatively identified” unless both diagnostic regions are simultaneously resolved. This cautious approach reflects the intrinsic limitations of neat-ice spectroscopy compared with matrix-isolation experiments, where molecular dilution yields sharper and more readily identifiable vibrational bands.

CH_3_NC has a characteristic N≡C stretching band, reported near 2165–2170 cm^–1^ in solid phases.^7,8^ A shoulder CH_3_CN around ∼2168 cm^–1^ becomes more intense at 80 K (see Figure 3e). This assignment is consistent withHudson & Moore (2004),^7^ who observed the emergence of the CH_3_NC band at 2170 cm^–1^ upon radiolyzing pure CH_3_CN ice as did Carvalho et al.^8^ We note that CH_3_NC is less stable than CH_3_CN, lying approximately 2.7 eV higher in energy, but can nevertheless be efficiently formed under energetic processing conditions. Although the isomerization barrier is high (about 4 eV), it remains below the localized energy deposited within ion tracks during irradiation, enabling nitrile-to-isonitrile conversion in the solid phase.^33^ These results indicate that methyl isocyanide (CH_3_NC) is formed in situ within the ice matrix through localized bond rearrangements rather than through thermal diffusion.

Swift Fe-ion irradiation promotes nitrile-to-isonitrile conversion more selectively than UV photons, consistent with the highly localized energy deposition characteristic of heavy-ion tracks, which favors direct molecular rearrangements. Once formed, subsequent chemistry in the ice may proceed through slower radical recombination and cage-confined processes on longer fluence scales. Consistently, Hudson and Moore^7^ showed that proton irradiation of CH_3_CN ices predominantly yields CH_3_NC, whereas UV photolysis produces both CH_3_NC and ketenimine (CH_2_C=NH).

Similarly, ion bombardment of CH_3_CN, reported a CH_3_NC:H_2_C=C=NH production ratio of ∼10:1, much higher than in UV photolysis (where CH_3_NC:H_2_C=C=NH ≈3:1).^33^ The heavy Fe ion projectiles likely follow this trend of preferential isonitrile formation. Although the CH_3_NC IR band is not very intense in the obtained spectra, its presence is consistent with these prior findings. Methyl isocyanide has not yet been detected in interstellar ices, but it has been observed in the gas phase in hot cores and might be released from ices upon energetic processing.^7,34^

Ketenimine (also called cyanomethine, H_2_C=C=NH) is another acetonitrile isomer, which can be viewed as the hydrogen migration product of acetonitrile (moving an H from the methyl group onto the nitrogen). Its IR signature is the C=N stretch mode, observed around 2030–2040 cm^–1^ in matrices.^6–8,26^ In the irradiated ice spectra, a small peak grows at 2035 cm^–1^, in excellent agreement with the literature value of 2034 cm^–1^ for H_2_C=C=NH. The formation of ketenimine likely proceeds via the radical intermediate ·CH_2_–C≡N plus an H atom, as follows: a CH_3_CN molecule loses an H from the methyl (forming ·CH_2_–C≡N), and that H immediately rebonds to the nitrogen of the −C≡N, yielding H_2_C=C=NH.^7^ This intramolecular mechanism is consistent with our observations.^7^ In pure CH_3_CN radiolysis, Hudson & Moore^7^ did not observe a strong ketenimine band (likely due to the dominance of the isonitrile pathway). However, soft X-ray experiments by Carvalho et al.^8^ did detect H_2_C=C=NH and even quantified its abundance relative to CH_3_NC.

The detection of a weak 2035 cm^–1^ peak confirms that a minor fraction of CH_3_CN converts to H_2_C=C=NH under Fe irradiation. Ketenimine is of astrochemical interest, as it has been tentatively identified in the interstellar medium (in Sgr B2) or at least suggested by modeling.^35^ As a consequence, the current laboratory results provide a solid-phase formation route for this species from a simple nitrile precursor. Figure 6 summarizes the fluence-dependent behavior of CH_3_CN destruction and product formation at both temperatures.

**Figure 6:**
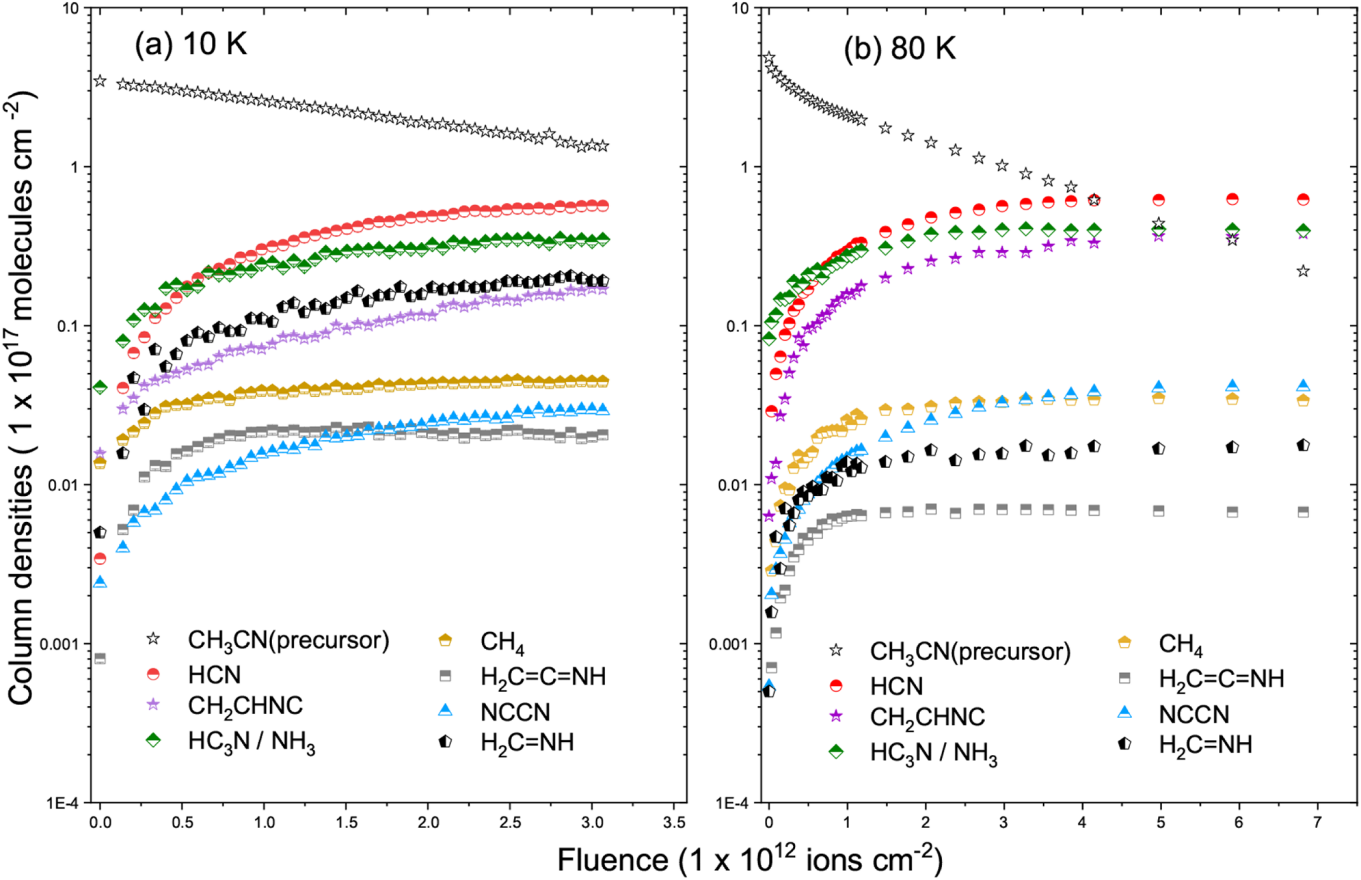
Column density evolution of radiolysis products formed from Fe-irradiated CH_3_CN ice as a function of ion fluence. Panels (a) and (b) show the results obtained at 10 and 80 K, respectively. The identified species include HCN, CH_2_CHNC, CH_4_, H_2_C=C=NH, NCCN, HC_3_N or NH_3_, and H_2_C=C=NH exhibiting distinct formation yields and saturation behaviors depending on the irradiation temperature.

#### Other Nitrile Products (C_2_H_5_CN, CH_2_CHCN, C_2_N_2_, HC_3_N)

The rich chemistry induced by heavy ions produces a variety of larger or smaller nitriles via radical recombination and fragmentation processes. One identified product is ethyl cyanide (C_2_H_5_CN). A new absorption band, at 1326 cm^–1^ grows up at high fluence, which may be assigned to the CH_2_ wagging mode of ethyl cyanide.^11^ This peak’s position matches the infrared spectra of solid CH_3_CH_2_CN (which has a strong feature near 1325 cm^–1^ in Figure 3c,f). The formation of ethyl cyanide likely proceeds by the recombination of a CH_3_ radical with a CH_2_CN radical. Notably, Hudson & Moore observed that in photolyzed acrylonitrile ices, features of CH_3_CH_2_CN appeared (via hydrogenation of the vinyl group).^11^

The detection of CH_3_CH_2_CN is significant because it is a known interstellar COM (detected in hot cores) and was also found as a photoproduct of CH_3_CN in UV experiments.^11^ Its presence here reinforces that carbon–carbon bond-forming reactions (radical recombination) occur even in the dense energy deposition tracks of heavy ions.

We have also searched for evidence of vinyl cyanide (acrylonitrile, CH_2_CHCN) itself. Acrylonitrile ice has a strong C≡N stretch at ∼2210–2230 cm^–1^,^7^ but this band occurs close to the parent CH_3_CN band. As the CH_3_CN is destroyed, we did not observe a clear new peak in that region, at 10 K (Figure 3b), suggesting that any CH_2_CHCN formed is quickly consumed (e.g., polymerized) or present in too low abundance to detect distinctly. Instead, we detected its isomer vinyl isocyanide (CH_2_CHNC) at 2136 cm^–1^, as can be seen at 80 K (Figure 3e). This implies that the −C≡N group of acrylonitrile preferentially rearranged to −N≡C in our experiment, consistent with the strong driving of nitrile-to-isonitrile conversion under anhydrous conditions.^7^ Indeed, Hudson and Moore^7^ assigned an IR feature to CH_2_CHNC in their nitrile radiolysis study, and we observe a similar feature. Vinyl isocyanide has not been observed in space, but our results suggest it could form in nitrile-rich ices subjected to cosmic rays.

Additionally, formation of the simplest dinitrogen species, cyanogen (NCCN), is possible via the combination of two CN radicals. We did not observe a distinct cyanogen absorption; for example, NCCN references are the IR-active mode near 2330 cm^–1^ since it is very close to the main band of acetonitrile (2252.1 cm^–^1). The weak band at 2063 cm^–1^ for NC–CN stretching was observed in our experiment (see Figure 2d), and a stronger band of NCCN was observed at 2341.3 cm^–1^ (see Figures 2c and 3b,e), same as seen by Hudson and Moore^7^ at 2345 cm^–1^.

Another potential product is cyanoacetylene (HC_3_N),^8^ which may be formed by loss of H_2_ from two acetonitrile molecules and bonding of the remaining fragments. We did not conclusively identify the HC_3_N fundamental (which lies around ∼2200 cm^–1^), but a combination band (ν_2_ + ν_4_) of HC_3_N might contribute to a shoulder observed around ∼3142 cm^–1^ ^1,8^ (Figure 3a,d). Overall, while heavier nitriles (with more than two carbon atoms) are minor products in pure CH_3_CN radiolysis, their formation cannot be entirely ruled out. The dominant pathways clearly favor one- and two-carbon species under our experimental conditions.^4,8^

#### Formation of N–H-Containing Species

A broad absorption centered at ∼3212 cm^–1^ is observed in irradiated samples for both temperatures, 10 and 80 K (Figure 3a,d). The band becomes significantly more intense and better defined at 80 K, indicating enhanced formation or stabilization of N–H–bearing products upon warming. This feature could be primarily attributed to the N–H stretching modes of solid ammonia (NH_3_), typically observed between 3211 and 3375 cm^–1^ in laboratory ices.^24,32^ A contribution from the C–H stretching vibration of cyanoacetylene (HC_3_N), which absorbs near 3300–3200 cm^–1^,^1^ cannot be excluded as a plausible molecule for the radiolytic product of CH_3_CN.

Alternatively, the simultaneous growth of weaker bands (Figures 1 and 2) around 1680 and 950 cm^–1^ may indicate a minor presence of *ketenimine* (H_2_C=C=NH), whose characteristic modes occur at ν­(N–H) ≈ 3200 cm^–1^, ν­(C=N) at 1670–1690 cm^–1^, and δ­(NH) at 940–960 cm^–1^,^5,7,36^ (Figures 1 and 2). Therefore, the 3212 cm^–1^ feature likely arises from overlapping contributions of NH_3_, HC_3_N, and possibly H_2_C=C=NH, reflecting the complex nitrogen chemistry induced by ion irradiation of CH_3_CN ice.

At 80 K, the enhanced molecular mobility within the ice matrix significantly increases the efficiency of radical diffusion, recombination, and structural rearrangement processes. Under these conditions, the radiolysis of CH_3_CN by swift heavy ions promotes both fragmentation and intramolecular transformations, enabling chemical pathways that are strongly suppressed at lower temperatures.

One of the characteristic products observed is ketenimine (H_2_C=C=NH), an isomer of acetonitrile of considerable astrochemical relevance. Instead, the well-established *intramolecular rearrangement* of CH_3_CN can be eq 3:

3 $${\mathrm{CH}}_{3}\mathrm{CN}\to H_{2}C=C=\mathrm{NH}$$

a pathway previously reported in UV, soft X-ray, and electron-induced chemistry of nitriles in cryogenic matrices.^6–8^ This transformation preserves the N–C connectivity of the parent nitrile while converting the C≡N triple bond into an imine (C=N) functional group.

The simultaneous detection of H_2_C=C=NH, NH_3_, and HC_3_N in the irradiated samples at 80 K indicates that both hydrogenation reactions and structural rearrangements are efficient under heavy-ion bombardment. Hydrogenation of small radicals (e.g., H + NH_2_ → NH_3_, H + CH_3_ → CH_4_) becomes more effective as thermal mobility increases, whereas rearrangement pathways such as CH_3_CN → H_2_C=C=NH are activated by local energy deposition and facilitated by the relaxation dynamics within the warmer ice.

These findings are consistent with earlier studies of CH_3_CN photoprocessing in mixed ices, which also report the concurrent formation of imines, amines, and longer nitrile chains.^6–8^ In the context of interstellar ice chemistry, the temperature dependence observed here suggests that regions experiencing transient heating or energetic particle fluxes may favor the emergence of nitrogen-bearing complex organic molecules through a combination of radical-driven and rearrangement-driven mechanisms.

No clear evidence for CH_3_NH_2_ (methylamine) formation was observed for the sample at 80 K, since its characteristic N–H stretching near 3300 cm^–1^ and C–N stretching around 1130 cm^–1^ are absent or obscured by overlapping CH_4_ and polymeric features.^8^ Therefore, the dominant nitrogen-bearing products under our conditions appear to be NH_3_, HC_3_N, and possibly H_2_C=C=NH, rather than fully hydrogenated amines. The identification of these species is astrochemically significant because they represent key intermediates linking simple nitriles (CH_3_CN, HCN) to amino and amide precursors detected in interstellar and circumstellar environments.^37^

#### Methane (CH_4_)

Another prominent new product is methane, identified by its strong ν_4_ deformation band at 1303 cm^–1^ (7.67 μm). This absorption band grows markedly with fluence, indicating efficient generation of CH_4_ in the irradiated ice. The band position is consistent with literature (solid CH_4_ ∼1302–1308 cm^–1^).^14,38^ Methane likely arises from recombination of radiolytic fragments of the methyl group. As CH_3_CN dissociates, ${\mathrm{CH}}_{3}^{•}$ radicals are released; these can capture H atoms to form CH_4_.^4^ Alternatively, two CH_3_ radicals may recombine to form ethane (C_2_H_6_), but in the current IR spectra we found no clear ethane features (e.g., the 820 cm^–1^ band of C_2_H_6_ is absent or very weak). These facts suggest that H atoms (which are plentiful from C–H bond dissociations) preferentially hydrogenate CH_3_ to CH_4_ before radical–radical coupling occurs. The formation of CH_4_ in the current measurements is in line with previous photochemical studies: soft X-ray irradiation of CH_3_CN at 13 K produces significant CH_4_ in the ice,^8^ and proton irradiation of nitrile ices has been reported CH_4_ as a minor product.^4^ Using the *A*_*v*_(CH_4_) as 6.4 × 10^–18^ cm molecule^–1^,^6^ the final CH_4_ column density in the current 80 K experiment is ∼3.4 × 10^15^ cm^–2^ (10 times less than HCN). Some fraction of the methane may reside in the ice or possibly outgas during warm-up; in an astrophysical context, CH_4_ could be released to the gas phase upon cosmic-ray heating of grains.

#### Polymeric Refractory Residue (HCN Polymers)

At the highest ion fluences, the spectra exhibit a rising baseline and broad absorbances between 2200 and 1300 cm^–1^ that cannot be attributed solely to discrete molecular species. These features indicate the formation of an IR-active refractory residue, a polymeric network enriched in C–N and N–H functional groups. Radiation-induced polymerization is a well-established outcome in nitrile ices,^7^ which often yield an ill-defined solid consistent with polyimine or polycyanide materials.

In the current experiments, the attenuation of sharp molecular bands and the concurrent growth of a broad continuum confirm the formation of such macromolecular species. HCN, abundantly produced during CH_3_CN radiolysis, can readily self-polymerize even at cryogenic temperatures, forming oligomers and polymers with conjugated C=N linkages (e.g., aminomalononitrile, diaminomaleonitrile) that absorb broadly in the 2100–1600 cm^–1^ region.^39^ The presence of NH_3_ or NH_2_ fragments may further promote acid–base interactions (e.g., ${\mathrm{NH}}_{4}^{+}{\mathrm{OCN}}^{-}$ ), contributing to the observed continuum.

After heavy irradiation, the samples display a visible yellow–brown deposit on the substrate, characteristic of complex organic refractory matter. Polymeric HCN is particularly relevant in this context, as it is regarded as a prebiotic precursor capable of yielding amino acids and nucleobases upon hydrolysis.^39^ Our results thus support the view that cosmic-ray processing of simple nitriles can produce *tholin*-like organic residues rich in C–N bonds,^40,41^ bridging laboratory astrochemistry with the nitrogen-rich aerosols observed in Titan’s atmosphere and with refractory organics found on icy grains in protostellar environments.

From an astrochemical perspective, these results demonstrate that transient heating or energetic-particle processing of nitrile-rich ices can strongly modulate molecular complexity by tuning the balance between radical trapping and diffusion-controlled chemistry.

#### IR-Derived Amorphization Cross Section from the ν­(C≡N) Band at 80 K

The irradiation-induced amorphization of CH_3_CN ice at 80 K was quantified using an infrared-derived crystallinity index (CI) extracted from the ν­(C≡N) stretching band, which is particularly sensitive to the local molecular environment. The CN band was decomposed into narrow and broad components associated with crystalline and amorphous environments, respectively. Those contributions have been deduced following approaches widely used to monitor irradiation-induced disordering in molecular ices using infrared spectroscopy.^42,43^ The crystallinity index is defined as the fractional crystalline contribution, by eq 4:

4 $$CI=\frac{A_{\mathrm{cryst}}}{A_{\mathrm{cryst}}+A_{\mathrm{am}}}$$

where *A*_cryst_ and *A*_am_ are the integrated absorbances of the crystalline and amorphous components. The CI was subsequently normalized to its initial value, yielding *CI*_norm_(*F*) (Figure 7b).

**Figure 7:**
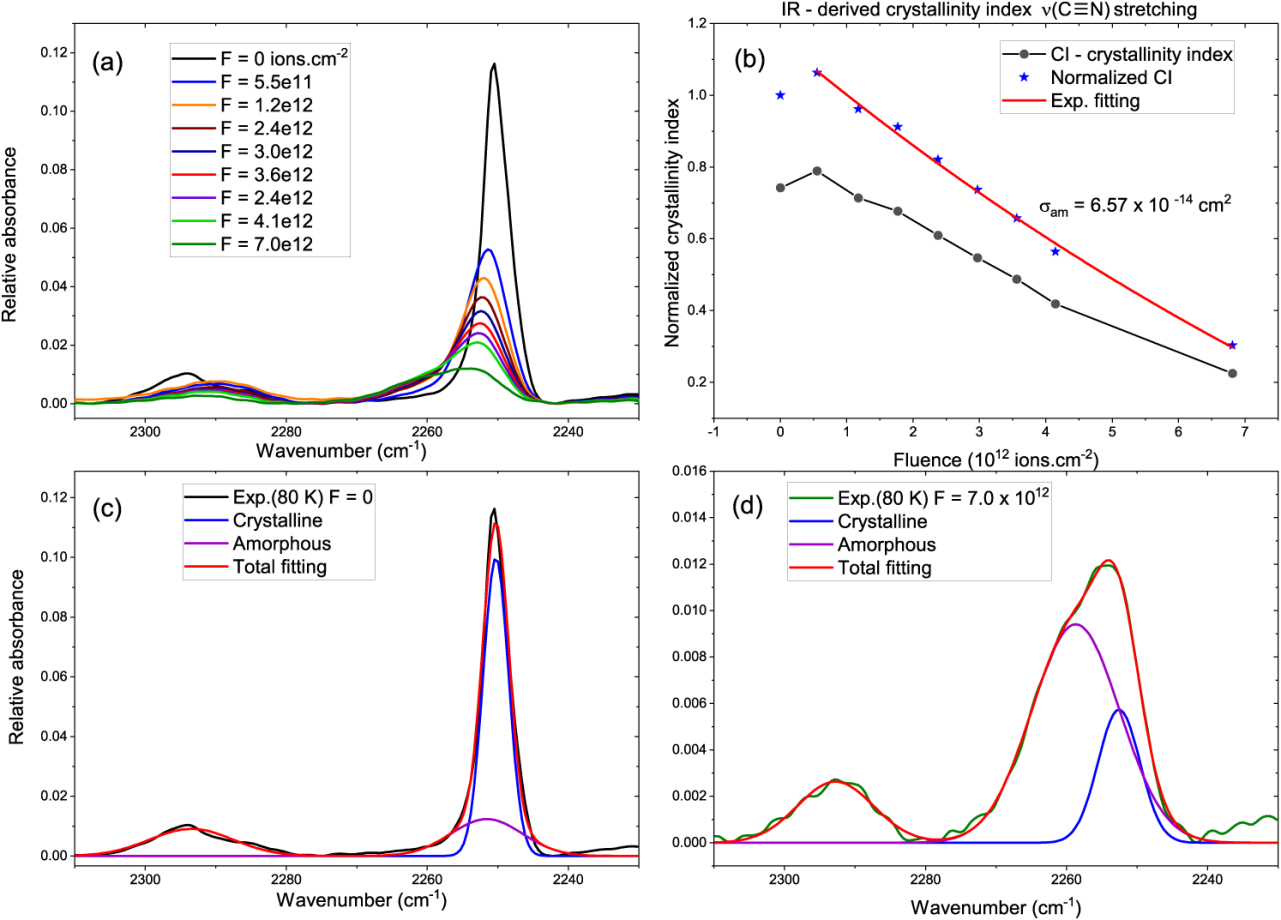
Fluence-dependent amorphization of CH_3_CN ice at 80 K monitored through the ν­(C≡N) stretching region. (a) Evolution of the CN band profile with increasing Fe-ion fluence. (b) IR-derived crystallinity index (CI, black) and normalized CI (red) as a function of fluence. (c) Representative decomposition of the CN band at *F* = 0, dominated by the crystalline component. (d) Decomposition at high fluence (*F* = 7.0 × 10^12^ ions cm^–2^), where the amorphous component prevails. Dark green line is the spectrum as acquired; blue and purple standards to crystalline and amorphous (a) and (c) respectively, red is the fitting of the spectrum using (a) and (c) components.

The fluence dependence of the normalized crystallinity index was modeled, assuming first-order kinetics for ion-induced amorphization using the eq 5.

5 $$CI_{\mathrm{norm}}\left(F\right)=\exp\left(-\sigma_{\mathrm{am}}F\right)$$

where *F* is the ion fluence (ions cm^–2^) and σ_am_ is an effective, IR-derived amorphization cross section. An exponential fit to the normalized CI data shown in Figure 7b yields

6 $$\sigma_{\mathrm{am}}=6.57\times 10^{-14}{\;\mathrm{cm}}^{2}$$

This value represents an operational spectroscopic measure of the rate at which crystalline order is lost under swift Fe-ion irradiation, defined here by the progressive disappearance of the crystalline contribution to the ν­(C≡N) band and the concomitant growth of a broadened, slightly red-shifted CN profile. It should be emphasized that σ_am_ (eq 6) is a band-specific, IR-derived quantity and does not correspond to a bulk crystallographic amorphization cross section obtained from diffraction techniques.^7,44^

In Figure 7a, the gradual broadening and intensity redistribution of the ν­(C≡N) band with fluence reveals the structural transformation of the ice. The corresponding crystallinity index shown in Figure 7b quantifies this trend, while the spectral decompositions at zero fluence and at *F* = 7.0 × 10^12^ ions cm^–2^ (Figure 7c,d) demonstrate the transition from a crystalline-dominated to an amorphous-dominated CN environment. Note that the normalized crystallinity index slightly exceeds unity at the lowest fluences (Figure 7b). This behavior does not indicate radiation-induced crystallization.^10,45,46^ Instead, it reflects early stage structural relaxation and spectral redistribution within the partially ordered ice, whereby minor band narrowing and intensity rebalancing can transiently increase the relative contribution assigned to the crystalline component during spectral decomposition. Such initial relaxation effects have been reported previously for ion and photon-processed molecular ices and are commonly observed when amorphization is tracked through spectroscopic proxies rather than long-range structural probes.

Finally, it is observed that σ_am_ is lower than the molecular CH_3_CN destruction cross section derived from band area loss, indicating that lattice disordering and chemical bond rupture proceed on different characteristic fluence scales, as previously observed in other nitrile- and oxygen-bearing ices irradiated by swift heavy ions.^47,48^ While parent-molecule destruction is governed primarily by local electronic excitation and dissociation within the ion tracks, amorphization reflects the cumulative loss of short and intermediate-range order sensed by the CN stretching mode. Together, these complementary cross sections provide a coherent picture of radiation-induced structural and chemical evolution in nitrile-rich astrophysical ice analogs.

### Determination of the Destruction Cross Section

The molecular destruction cross section (σ_*d*_) for the precursor CH_3_CN and the formation cross sections (σ_*f*_) of its products are derived from the variation of the infrared band areas as a function of ion fluence (*F*). For each selected absorption band, the integrated area *S*(*F*) was converted to the column density *N*(*F*) using the Beer–Lambert relation shown in eq 7:

7 $$N\left(F\right)=\frac{\ln\left(10\right)\;S\left(F\right)}{A}$$

where *A* is the band strength (in cm molecule^–1^) and ln(10) converts absorbance from base-10 to natural logarithms. The adopted *A* values for CH_3_CN and its radiolysis products are listed in Table 3.

**Table 3:** Band Strengths *A_v_* Adopted for Column Density Calculations of the New Species Formed during Fe-Ion Irradiation of Acetonitrile Ice^a^

Species	Mode/Assignment	Position (cm^–1^)	*A_v_* (cm molecule^–1^)
**CH**_**4**_	ν_4_ C–H band	1303 ^6,25,26^	6.4 × 10^–18^
(CH_3_)CH_2_CN	(C≡N) CH_2_ wag	1326^b^ ^11^	–
**H**_**2**_ **C=NH** or	ν_2_ (C=C/C=N stretch)	1645 ^26–28^	3.8 × 10^–18^
CH_3_CH=NH^29–31^	ν(C=N)		–
**H**_**2**_ **C=C=NH**	ν_3_ (C=C=N stretch)	2033 ^6–8,26^	7.2 × 10^–17^
**HCN**	ν(C≡N)	2086 ^2,6,8^	5.1 × 10^–18^
**CH**_**2**_ **CHNC**	C≡N stretch (ν(C≡N))	2138 ^1,8^	^c^4.2 × 10^–18^
(CH_3_)_3_CNC	C≡N stretch	2140 ^7^	–
CH_3_NC^b^	ν(C≡N)	2165 ^7,8^	2.2 × 10^–18^
NCCN	ν_3_ (C≡N stretch)	2342 ^7^	–
CH_3_NC^7^ or **NCCN** ^1^		2167	^c^6.3 × 10^–20^
HC_3_N	ν_2_+ν_4_	3138 ^8^	–
**NH**_**3**_ or	ν_1_,ν_*s*_ N–H stretch	3212 ^26,32^	2.2 × 10^–17^
HC_3_N^1^	ν_1_ C–H stretch		^c^7.1 × 10^–17^

a The molecules that we use to determine the cross sections are indicated in bold.

b Observed only at 80 K.

c Calculated from integrated absorption coefficients.^1^

Assuming a first-order decay process, the evolution of the precursor column density with fluence is expressed as

8 $$N\left(F\right)=N_{0}\;\exp\left(-\sigma_{d}F\right)$$

where *N*_0_ is the initial column density (prior to irradiation) and σ_*d*_ is the apparent destruction cross section (in cm^2^). Apparent destruction cross section means that it corresponds to the sum of radiolysis and sputtering effects.^49^ Thus, σ_*d*_ is obtained from the slope of the linear fit of ln­[*N*(*F*)/*N*_0_] versus *F*. Similarly, for newly formed species the growth curves are determined by fitting the complementary exponential form:

9 $$N_{j}\left(F\right)=N_{j\infty }\left[1-\exp\left(-\sigma_{f,j}F\right)\right]$$

where *N*_*j,*∞_ represents the saturation column density of the *j* species reached at high fluence and σ_*f*,*j*_ the apparent formation cross section of the species.

This formalism has been widely applied in laboratory astrochemistry to quantify molecular processing in ices under energetic irradiation. In particular, Carvalho and Pilling^8^ employed the same approach to determine destruction and formation cross sections in CH_3_CN ices irradiated by soft/tender X-rays (6–2000 eV). In the present work, the same approach was applied to analyze the ices irradiated by swift heavy ions, allowing direct comparison between low and high linear energy transfer (LET) regimes.

### Temperature Effects on the Radiolysis Chemistry (10 K vs 80 K)

At 10 K, radiolysis products remain largely trapped within the rigid ice matrix, where radical diffusion is strongly suppressed and chemical evolution is dominated by local energy deposition within Fe-ion tracks, favoring intramolecular isomerizations such as CH_3_NC and H_2_C=C=NH/CH_3_C=NH. At 80 K, the increased mobility of H atoms and light radicals accelerates CH_3_CN fragmentation, leading to a significantly higher effective destruction cross section for the parent molecule relative to 10 K. This trend has previously been observed in other molecular ices, including aromatic nitriles such as pyridine, and was quantified by Prudence Ada Bibang et al., who demonstrated that molecular destruction cross sections systematically rise around 80 K due to increasing lattice disorder and radical-mediated reaction pathways.^48^

Discrete irradiation steps show that crystalline CN band splitting persists up to ∼7 × 10^11^ ions cm^–2^, but collapses into a single, broadened, slightly red-shifted C≡N profile at fluences above 3 × 10^12^ ions cm^–2^, marking the regime where long-range lattice order is effectively lost. Although the qualitative set of radiolysis products (e.g., HCN, CH_4_, nitrile isomers) is similar at both temperatures, their relative yields and reaction progression differ substantially. Minor products identified include cyanoacetylene (HC_3_N) via C–H stretching in the 3300–3200 cm^–1^ region and its C≡N stretch at 2165 cm^–1^, cyanogen (NCCN) at 2341 cm^–1^, H_2_C=NH at 1645 cm^–1^, and CH_2_CHNC at 2138 cm^–1^, confirming that both imine and isonitrile/isonitrile-bearing channels operate concurrently under Fe-ion tracks. These assignments agree with Figures 8 and 9 and with previous laboratory reports on radiation-processed nitrile ices.^1,5,7^

**Figure 8:**
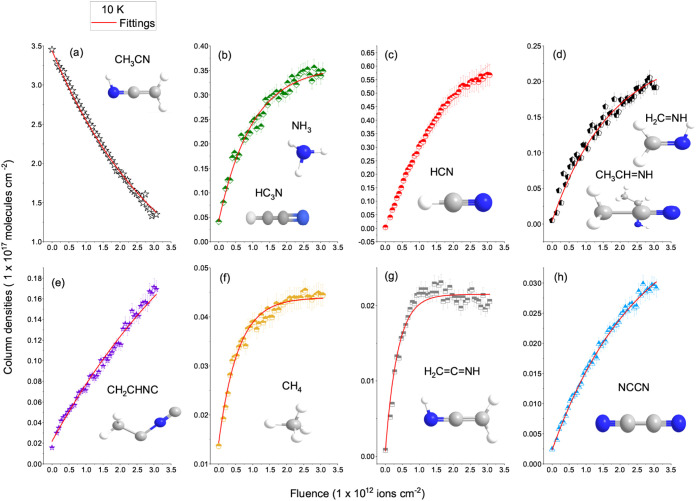
Evolution of column densities of CH_3_CN and its product species as a function of Fe ion fluence at 10 K. Experimental data points (symbols) were fitted with exponential functions (red lines) to derive apparent destruction and formation cross sections according to eqs 8 and 9 respectively. Panel (a) shows the decay of the precursor CH_3_CN, while panels (b–h) present the growth of the main radiation products: NH_3_ or HC_3_N, HCN, H_2_CNH or CH_3_CH=NH, CH_2_CHNC, CH_4_, H_2_C=C=NH and NCCN, respectively. Column densities are expressed in units of 1 × 10^17^ molecules cm^–2^.

**Figure 9:**
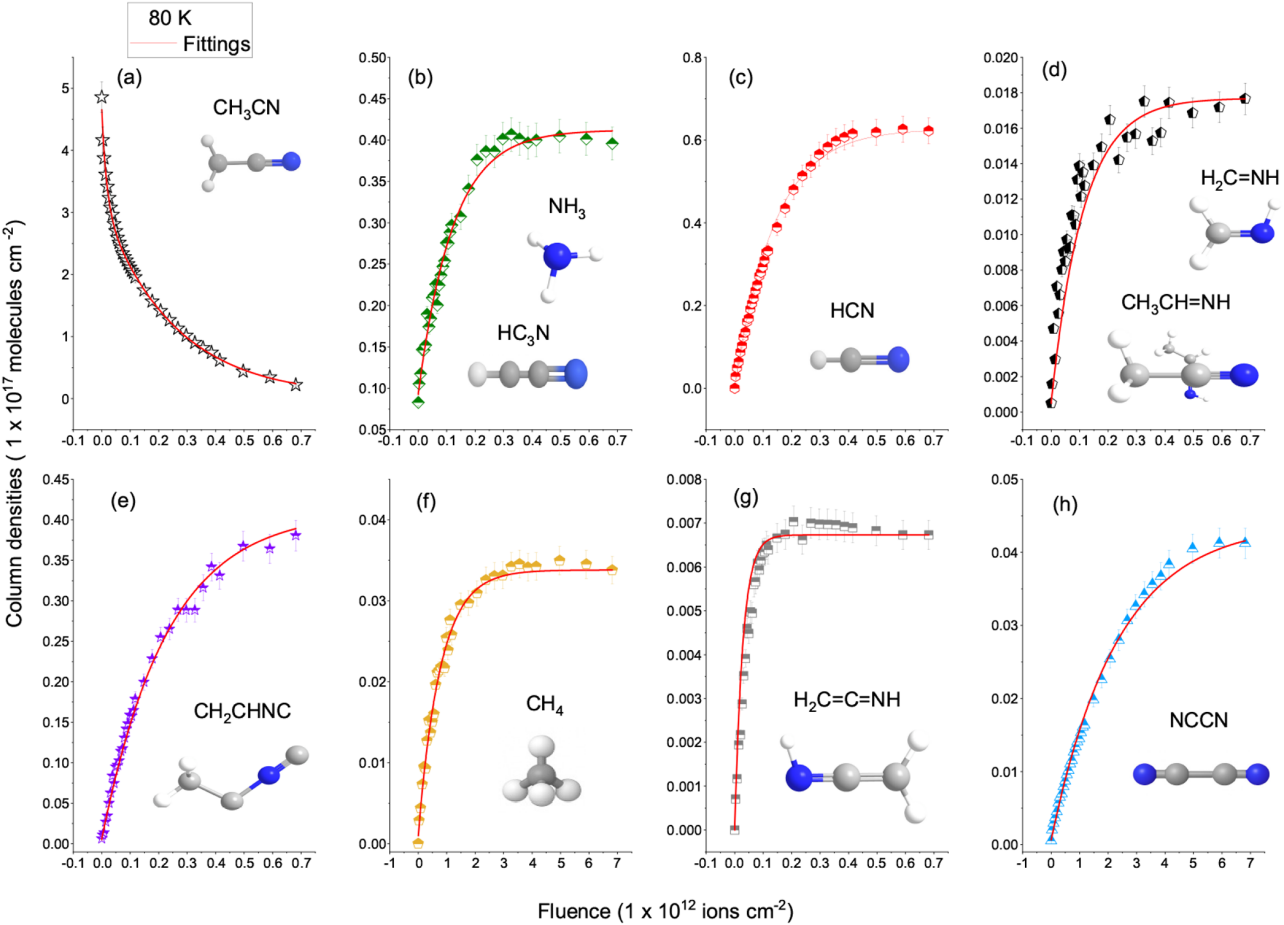
Evolution of column densities of precursor and products species formed after Fe irradiation of CH_3_CN ice at 80 K as a function of ion fluence. Symbols represent experimental data and red lines the exponential fittings used to derive apparent destruction and formation cross sections according to eqs 8 and 9 respectively. Panel (a) shows the decay of CH_3_CN, while panels (b–h) display the formation of major products: NH_3_ or HC_3_N, HCN, H_2_CNH or CH_3_CH=NH, CH_2_CHNC, CH_4_, H_2_C=C=NH and NCCN, respectively. Column densities are expressed in units of 1 × 10^17^ molecules cm^–2^.

At 10 K, initial radiation steps produce a trapped population of radicals and metastable products, with limited diffusion. Many reactive intermediates (H atoms, CH_3_, CN, etc.) will recombine locally or become immobilized in the matrix cage if they do not encounter a partner immediately. The formation of CH_3_CH_2_CN was less evident at 10 K, presumably because the CH_2_CN and CH_3_ fragments were not free to diffuse together. The 10 K spectra showed relatively stronger CH_3_NC and H_2_C=C=NH features (in proportion to HCN) compared to 80 K, suggesting that intramolecular isomerizations (which occur in situ upon energy deposition) dominate at low temperature, whereas at 80 K some of those −CN fragments instead travel and form other products (such as HCN or polymer). Table 4 summarizes the apparent destruction cross section of CH_3_CN and the apparent formation cross sections of the main products at 10 K and 80 K, derived from fits to the fluence-dependent curves.

**Table 4:** Apparent Cross Sections σ Derived from the Fitted Curves (Red) in the 10 K (Figure 8) and 80 K (Figure 9)

Species	Main band (cm^–1^)	σ (10 K) (× 10^–12^ cm^2^)	σ (80 K) (× 10^–12^ cm^2^)
	Precursor	destruction cross section	
CH_3_CN	2252	2.3 ± 0.8	5.6 ± 1.0
	Products	formations cross sections	
HC_3_H or NH_3_	3212.0	0.93 ± 0.17	0.82 ± 0.15
HCN	2086.2	0.42 ± 0.12	0.72 ± 0.14
H_2_C=NH	1645.4	0.57 ± 0.10	0.88 ± 0.16
CH_2_CHNC	2137.7	0.15 ± 0.05	2.2 ± 0.9
CH_4_	1303.2	1.4 ± 0.7	1.6 ± 0.7
H_2_C=C=NH	2033.5	1.7 ± 0.8	3.4 ± 1.0
NCCN	2341.3	0.22 ± 0.10	0.43 ± 0.12

By contrast, at 80 K the ices are in a regime where the smallest radicals (H, possibly CH_3_) have appreciable mobility. Hydrogen atoms in particular can quantum tunnel or thermally hop even at tens of kelvin; thus 80 K promotes hydrogen addition reactions throughout the ice. It also explains why CH_4_ grew more rapidly and to a higher end concentration in the 80 K run: mobile H atoms could find and hydrogenate CH_3_ radicals more efficiently. Increased mobility at 80 K also means that once a radical like CN is formed, it might travel further before recombination, enabling cross-linking between tracks. This potentially enhances polymerization at 80 K, indeed, the 80 K-irradiated ice showed a more pronounced residual absorption (polymer) than the 10 K case for the same fluence, consistent with radicals migrating and reacting to form larger networks. Warmer ice also undergoes gradual structural relaxation; CH_3_CN ice at 80 K is closer to a supercooled liquid state than the rigid amorphous solid at 10 K.^6,7^ This softening could facilitate molecular rearrangements and encounters between reaction intermediates.

Another difference is that some volatile products (like CH_4_, H_2_) might diffuse out or desorb more readily at 80 K. We observed that, after higher fluences, the 80 K ice showed a slight loss of the CH_4_ band area relative to what would be expected if all remained in the ice, hinting that a portion may have evaporated (80 K is below the CH_4_ sublimation point, but ion energy deposition can induce spot-heating and localized desorption). At 10 K, virtually all products remained trapped. This difference could affect the eventual ice composition that survives; e.g., 80 K irradiation might deplete the ice of the most volatile species (CH_4_, H_2_, and even HCN in part) over time, whereas 10 K irradiation keeps them frozen in. From an astrochemical perspective, 10 K corresponds to the interiors of dense clouds where all products accumulate in the ice, while 80 K might resemble a warmer grain or ice on an airless body where some radiolysis gases can escape.

In summary, the 80 K irradiation leads to a more processed, polymer-rich residue with additional hydrogenation products (NH_3_) and potentially slightly lower relative yields of the intramolecular isomers compared to the 10 K case. Nonetheless, the key products HCN, CH_4_, and secondary nitriles such as HC_3_N and NCCN appear in both cases, underscoring their robust formation pathways. The temperature-dependent behaviors reported in this work are in line with previous studies: for instance Mouzay et al.^10^ noted small shifts and intensity changes in CH_3_CN IR bands as ices are warmed and Hudson and Moore^7^ emphasized that certain reactions (like isonitrile formation) occur in “dry” conditions but are quenched when more mobile species (like H_2_O) are present.

Here, mobility comes from temperature rather than a solvent matrix, but the effect is analogous, increased flexibility of the medium allows alternative chemistry (hydrogenation, radical–radical combination) to compete with direct intramolecular conversions.

Therefore, the product distribution reported here is not expected to translate quantitatively to realistic interstellar ice mixtures, but it provides a mechanistic framework for understanding how nitrile chemistry responds to energetic processing under different matrix conditions. In mixed astrophysical ices, CH_3_CN radiolysis is likely to contribute indirectly to the ubiquitous 4.62 μm “XCN” feature through OCN^–^ formation, while the direct detection of solid isonitriles may be restricted to environments where oxygen-bearing species are depleted or where rapid desorption preserves transient products.

## Astrochemical Implications

It is important to emphasize that the present experiments were performed with neat CH_3_CN ice, whereas in astronomical environments acetonitrile is expected to be a minor constituent embedded within more abundant ice matrices dominated by H_2_O, CO, and CO_2_. Observational constraints indicate that solid CH_3_CN occurs, at most, at the percent level relative to H_2_O in interstellar ices.^50^ As a result, the chemistry observed in pure CH_3_CN ice should be regarded as an end-member case, useful for isolating intrinsic reaction pathways, identifying primary products, and establishing reference infrared fingerprints for nitrile radiolysis.

Previous laboratory studies have shown that matrix composition strongly influences the outcome of nitrile irradiation. In H_2_O-rich ices, CN-bearing systems tend to favor the formation of OCN^–^ through reactions involving HNCO and proton transfer, while the production of detectable isonitriles (e.g., CH_3_NC) and ketenimine species is strongly suppressed.^5,7,11^ By contrast, in oxygen-poor or CO-rich matrices, where oxidation pathways are limited, intramolecular isomerization and nitrile-to-isonitrile conversion become more competitive, leading to enhanced yields of species such as CH_3_NC and H_2_C=C=NH.

The current experimental results demonstrate that heavy-ion cosmic rays can drive a rich chemistry in acetonitrile-rich ices, converting a simple CN-bearing molecule into a variety of smaller molecules (HCN, CH_4_, NH_3_), isomeric species (CH_3_NC, H_2_C=C=NH), and complex polymeric material. In this context, it is instructive to compare the present heavy-ion irradiation results with recent X-ray radiolysis studies of acetonitrile performed under matrix-isolation conditions. Kameneva et al.^18^ and Volosatova et al.^19^ investigated the radiation-induced transformations of isolated CH_3_CN molecules trapped in noble gas matrices using X-ray irradiation and FTIR spectroscopy, providing high-resolution spectra and a detailed identification of primary and secondary products. The use of inert matrices allowed narrow vibrational features to be resolved and minimized secondary reactions, yielding reliable reference data for band positions and product assignments.

Key species observed in the present work, including HCN, CH_4_, ketenimine-related products, and CH_3_NC, are consistent with those reported in the X-ray/matrix-isolation studies, lending support to our spectral assignments despite the broader bands and stronger overlap inherent to neat condensed ices. Differences in relative yields and reaction sequences arise primarily from environmental effects: in matrix-isolated systems, molecular dilution stabilizes primary fragments and suppresses radical–radical recombination, whereas in neat ices the high molecular density favors secondary reactions, recombination, and polymer growth.

Although the irradiation sources differ (swift heavy ions versus X-rays), the elementary chemistry in both cases is largely governed by low-energy secondary electrons, since heavy ions predominantly lose energy through electronic stopping. Quantitative differences in product distributions are therefore attributed to differences in linear energy transfer (LET) and spur density: heavy ions generate highly localized excitation tracks that enhance condensed-phase chemistry, whereas X-ray irradiation deposits energy more sparsely, favoring isolated fragmentation pathways.^18,19^

The comparison between neat condensed films and matrix-isolated molecules highlights the strong influence of the molecular environment on radiation-induced chemistry. In astrophysical ices, organic molecules such as CH_3_CN are unlikely to form extended pure films, but are more commonly dispersed within dominant matrices composed of H_2_O, CO, and CO_2_. Matrix isolation therefore represents an extreme case of molecular dilution, while neat ices represent the opposite limit of maximal molecular proximity. Together, these two approaches provide complementary end-member scenarios that are both relevant for astrochemistry: isolated molecules allow accurate identification of intrinsic reaction channels, whereas compact ices capture the collective effects of radical diffusion, recombination, and polymer formation expected in dense grain mantles.

It was explicitly demonstrated in matrix-isolation X-ray radiolysis experiments that prolonged irradiation of isolated CH_3_CN molecules at cryogenic temperatures leads to efficient dehydrogenation and the formation of CCN and CNC radical species, which undergo interconversion and may reach a stationary population under continued irradiation.^19^ In the present swift heavy-ion irradiation experiments of neat CH_3_CN films, no distinct infrared features could be unambiguously assigned to free CCN or CNC radicals. This nonobservation does not imply that such species are not formed as transient intermediates, but rather reflects the strong influence of the molecular environment and irradiation conditions. In inert matrices, radical diffusion and bimolecular recombination are suppressed, allowing radicals to accumulate and remain spectroscopically observable. By contrast, in compact molecular films irradiated by swift heavy ions, the high local excitation density within ion tracks promotes rapid radical–radical recombination, hydrogen abstraction, and polymer growth, reducing the steady-state concentration of free radicals below the detection limit of FTIR spectroscopy. In addition, potential CCN/CNC absorptions fall in spectral regions that partially overlap with intense bands from the parent molecule and other radiolysis products in neat CH_3_CN ice. We therefore interpret CCN and CNC as plausible short-lived intermediates that are efficiently consumed by secondary reactions in dense condensed phases, rather than as stable end products. In this context, the formation and rapid consumption of CCN and CNC radicals can be naturally understood within a spur-chemistry framework, in which the high local density of excitations and radicals produced along swift heavy-ion tracks strongly favor ultrafast recombination and secondary chemistry over the accumulation of isolated radical species.

This has several implications for astrochemistry. First, CH_3_CN, which is known to be present in interstellar ices only in trace amounts (upper limits of a few percent relative to H_2_O), could still act as a progenitor of abundant HCN in the solid phase.^50^ If cosmic rays penetrate dense cloud cores, they could continuously produce HCN in situ from whatever CH_3_CN is available, even if CH_3_CN itself is not easily observable in the ice.^7^

Upon sublimation or desorption, this HCN could contribute to the gas-phase HCN seen in star-forming regions. Second, the efficient formation of CH_4_ indicates that even in oxygen-poor ices, organics can generate methane under energetic processing. This may be relevant to Titan’s atmospheric chemistry: Titan’s N_2_/CH_4_ atmosphere is replenished by CH_4_ from the surface, and one proposed source is cosmic-ray chemistry converting solid organics to CH_4_. The current results show that a solid nitrile can indeed yield CH_4_ under MeV ion impact, supporting this hypothesis in principle.

From an astrochemical perspective, it is essential to distinguish radiation fields not only by their fluxes but also by their energy deposition characteristics. In environments such as Titan’s upper atmosphere and surface, the flux of swift heavy ions is indeed much lower than that of solar UV photons, magnetospheric electrons, and keV ions.^51^ Consequently, heavy-ion irradiation is not expected to dominate surface chemistry in terms of global production rates. Instead, experiments such as the present one are best interpreted as process-oriented studies, aimed at identifying reaction pathways, product families, and cross sections under conditions of dense energy deposition, rather than as direct predictors of surface chemistry rates on Titan.

Nevertheless, swift heavy ions are characterized by very high linear energy transfer (LET), leading to dense ionization tracks, strong local excitation, and the formation of chemically rich spurs. In condensed molecular ices, a substantial fraction of the chemistry induced by all radiation types ultimately proceeds through low-energy secondary electrons; therefore, many elementary reaction mechanisms overlap between heavy ions, keV electrons, and VUV photons, while product yields and branching ratios can differ quantitatively as a function of LET.^43,52^ Heavy-ion irradiation thus probes a localized, high-dose chemistry regime that complements the more spatially dilute processing induced by photons and electrons.^44^

In astrophysical environments, heavy-ion processing is expected to be relevant primarily through the contribution of galactic cosmic rays, which include a minor but energetically significant heavy-ion component. Such processing becomes important (i) in dense and translucent interstellar clouds, where icy grain mantles are shielded from UV photons but exposed to cosmic rays over long time scales, (ii) in circumstellar and protoplanetary environments with attenuated UV fields, and (iii) in icy bodies and planetary surfaces where the large penetration depth of energetic ions allows radiation-induced chemistry to occur well below the surface.^47,53^

The production of nitrile isomers (CH_3_NC, etc.) in ices is intriguing. Gas-phase CH_3_NC has been detected in hot core regions, but its origin is debated. A solid-phase formation and prompt desorption are one possibility. The current data provide the first direct evidence that CH_3_NC can be synthesized in the ice by cosmic-ray analogs. Although we did not specifically drive desorption in our setup, cosmic-ray hits are known to cause sputtering; thus, some fraction of CH_3_NC formed might escape the ice promptly during bombardment. Future astrochemical models should include CH_3_NC formation from CH_3_CN under cosmic-ray bombardment as a potential pathway. The same holds for H_2_C=C=NH: while not yet confirmed observationally in ices, its facile formation in the current experiment means it could be present in radiation-processed grains and potentially released (ketenimine is quite reactive, but if stabilized in ice, it might accumulate).

Finally, the formation of an organic refractory residue from pure CH_3_CN is notable. Even a single-component ice can produce a complex “tholins-like” material under irradiation.^54^ In astrophysical contexts, ices are mixtures; nonetheless, the tendency of nitriles to polymerize implies that nitrogen from molecules like CH_3_CN can end up sequestered in large macromolecules on grains. These nitrile polymers (often compared to HCN-polymers) are of considerable astrobiological interest because of their potential to yield amino acids upon hydrolysis.^39^

Laboratory studies have shown HCN polymers can contain amine and amide functionalities and act as precursors to biomolecules.^39^ Therefore, cosmic-ray processing of interstellar ices containing CH_3_CN (and similar nitriles) could contribute to the inventory of complex organics in protostellar nebulae and on primitive Solar System bodies. For example, cometary matter and meteorites might contain residues that partially originate from nitrile radiolysis. Bernstein et al.^2^ and others have suggested that photolysis of mixed ices yields such polymeric N-rich residues which, when returned to liquid water, can form amino acids. Our work provides direct experimental evidence supporting this scenario via heavy ion processing.

In conclusion, the irradiation of acetonitrile ices by heavy ions at low temperatures leads to a rich chemistry: we observe the destruction of CH_3_CN and the formation of a suite of molecules (HCN, CH_4_, HC_3_N, CH_3_NC, H_2_C=C=NH, CH_2_=NH, C_2_H_5_N, NCCN, etc.) as well as amorphous polymeric material. These findings, supported by previous photochemical and radiolysis studies,^4,6–8^ highlighting the role of CH_3_CN as a potential progenitor of both simpler species and complex organic matter in astrophysical ices. Such laboratory data are invaluable for interpreting infrared observations of cosmic ices and for guiding astrochemical models of N-bearing organic synthesis in space. The comparison between 10 and 80 K conditions further emphasizes how physical conditions (temperature, matrix composition) can steer the chemistry toward different outcomes, an important consideration when extrapolating laboratory results to varied astronomical environments. The results here will aid in assigning IR spectral features in upcoming observations (e.g., JWST spectra of protostellar ices) by providing reference signatures for radiolysis products of acetonitrile and improve our understanding of the chemical pathways that link simple nitriles to complex prebiotic molecules in the universe.
